# A comparative evaluation of safety and efficacy of rosuvastatin, simvastatin, and atorvastatin in patients of type 2 diabetes mellitus with dyslipidemia

**DOI:** 10.4103/0973-3930.53124

**Published:** 2009

**Authors:** Samir Maruti Adsule, Mirza Shiraz Baig, P. R. Gade, P. N. Khandelwal

**Affiliations:** Department of Pharmacology, Govt. Medical College, Aurangabad, India

**Keywords:** Aatorvastatin, dyslipidemia, rosuvastatin, simvastatin, type-2 diabetes mellitus

## Abstract

**AIM:**

To evaluate and compare the safety and efficacy of rosuvastatin, simvastatin, and atorvastatin in patients of type 2 diabetes mellitus with dyslipidemia.

**MATERIALS AND METHODS:**

This open-label, randomized, parallel group, comparative, prospective study of 12-weeks duration included 60 patients of type-2 diabetes with dyslipidemia having good glycemic control with fixed dose combination of tablet glimepiride + metformin and divided into three groups of twenty each. Group-1 patients have received tablet rosuvastatin 10 mg once daily, group-2 received tablet atorvastatin 10 mg once daily, and group-3 received tablet simvastatin 10 mg once daily for 12 weeks each. The levels of serum cholesterol, serum triglyceride, LDL, VLDL, and HDL were assessed at baseline and at the end of 12 weeks.

**RESULTS:**

The mean serum cholesterol, serum triglyceride, LDLc, and VLDLc levels were significantly reduced on therapy (*P*<0.001). Simultaneously, the mean levels of HDL were highly significantly increased (*P*<0.001) after therapy for 12 weeks with rosuvastatin, atorvastatin, and simvastatin. Reduction of LDL levels in rosuvastatin group was statistically significant when compared with those of simvastatin group (*P*< 0.05) but was statistically nonsignificant when compared with atorvastatin group (*P*> 0.05). Conclusion: 10 mg of rosuvastatin was comparable to 10 mg of atorvastatin and more efficacious than 10 mg simvastatin in reducing LDL levels after 12 weeks of therapy in patients of type 2 diabetes mellitus with dyslipidemia.

## Introduction

Diabetes mellitus is a very commonly occurring metabolic disorder characterized by hyperglycemia and altered metabolism of lipids, proteins, and carbohydrates which is due to absolute or relative deficiency of insulin or insulin resistance.[1]

Diabetes mellitus is associated with increased oxidative stress due to hyperglycemia. The oxidative damage plays a role in development of micro and macro vascular complications, leading to a significant impact on quality of life. Long-term complications involve almost all vital organs such as heart, eyes, kidney, blood vessels, and nervous system. These complications lead to the development of obesity, hypertension, dyslipidemia, and insulin resistance.[2]

There is a close association between complications of diabetes and diabetic dyslipidemia. Diabetic dyslipidemia accounts for around 80 percent diabetic deaths due to cardiovascular complications. There is a growing body of evidence to show that hyperglycemia and dyslipidemia are associated with excess of cardiovascular risk.[3]

Treatment of type 2 diabetes requires the agents that act beyond their blood glucose effect. Drug therapy that not only has an effect on blood glucose level but also has a beneficial effect on dyslipidemia, hypertension, obesity, hyperinsulinemia, and insulin resistance is likely to be the most useful therapy in treating type-2 diabetes.[4]

Diabetic patients tend to have a higher concentration of small dense LDL particles, which are associated with higher CHD risk. Lowering LDL levels is the first priority in treating diabetic dyslipidemia. Statins are the first drug of choice, followed by resins or ezetimibe, then fenofibrate, or niacin. Current evidence and guidelines mandate that diabetic dyslipidemia should be treated aggressively, and lipid goals can be achieved in most patients with diabetes when all available products are considered and, if necessary, used in combination.[5]

Different statins require different dosing to reach the same LDL level. The lowering of LDL levels with statins varies from 20 to 60%. Therefore, the greatest effects are seen with the most potent statins such as simvastatin, atorvastatin, and rosuvastatin in the higher doses. Besides, majority of diabetic patients are at risk of coronary heart disease and deserve LDL cholesterol lowering to the currently recommended targets.[6]

The diabetes atorvastatin lipid intervention (DALI) study concluded that either 10 or 80 mg of atorvastatin is equally effective in the treatment of diabetic dyslipidemia.[7] Intensive lowering of LDL-C with high dose atorvastatin does not result in a significant reduction in the primary outcome of major coronary events, but reduces the risk of other composite secondary end points and nonfatal acute MI.[8]

Atorvastatin is more effective than simvastatin-based therapies in achieving treatment targets in patients with familial hypercholesterolemia.[9] Rosuvastatin 10 and 20 mg tablet improves the overall lipid profile of hypercholesterolemic patients better than does milligram equivalent doses of atorvastatin.[10]

Considering the above-mentioned facts, it seems that prevention of cardiovascular complications of diabetes must be considered as a national public health goal in the light of the increasing prevalence of the disease and the high frequency and seriousness of its complications.

The present study was thus planned to primarily evaluate and then to compare the efficacy and safety of newer emerging and promising statin rosuvastatin *vs* existing commonly used statins such as simvastatin and atorvastatin in patients with type-2 diabetes mellitus with dyslipidemia, so as to guide the present treatment strategies in the management of diabetes with dyslipidemia in Indian population.

## Materials and Methods

This study was open-label, randomized, parallel group, comparative, prospective study in patients with type 2 diabetes mellitus with dyslipidemia. Sixty patients of type-2 diabetes with dyslipidemia having good glycemic control with fixed dose combination of tablet glimepiride + metformin were included in the study after taking written informed consent. The exclusion criteria for patients were clinically significant deviation from normal in physical examination, laboratory parameters, ECG, or chest X-ray. Clinically significant cardiovascular disease, including a history of congestive heart failure, angina pectoris within 1 year and history of myocardial infarction within 1 year, convulsive disorder, clinically significant gastrointestinal disease, including active peptic ulcers within the preceding 5 years, renal disease, hepatic disease, hematologic disease and insulin-dependent diabetes mellitus, and known infection with human immunodeficiency virus, were excluded. Subjects with the presence of any acute illness, h/o sensitivity to statins, history of any musculo-skeletal disorder, history of alcohol, barbiturate, marijuana, or multidrug abuse, participation in other investigational drug studies within 30 days before the start of the study, subjects who are unlikely to be compliant with the protocol requirements, pregnant or lactating females, patients with history of use of any of the statins for at least 6 months prior to the commencement of the study and smokers were also excluded.

Approval of the ethical committee of Government Medical College and Hospital, Aurangabad was taken prior to the start of the study. Sixty patients were enrolled in the study after satisfying the inclusion and exclusion criteria. Included patients were explained in detail about the study protocol and related hazards. Informed written consent was obtained from all the patients. Those included underwent all baseline investigations such as liver function tests, kidney function tests, blood sugar level, fundoscopy, and baseline lipid profile, which was repeated at the end of the study. Enrolled patients were divided into three groups of twenty each by computer generated randomization chart (calculated from True Epistat, Standard version 1999). Group-1 patients received rosuvastatin10 mg tablet once in a day, group-2 received atorvastatin tablet 10mg once in a day, and group-3 received simvastatin tablet 10 mg once daily for a period of 12 weeks. Each patient in the respective group was provided with the drug supplies for fifteen days and was asked to visit the diabetic clinic for follow up and for collection of drugs. At each follow-up visit, patients were assessed for glycemic control, and history pertaining to adverse drug effects was asked. All patients were given advice about diet and exercise.

The primary objectives for the study were:

1. To evaluate the effect of rosuvastatin, atorvastatin, and simvastatin on the lipid profile of patients with type 2 diabetes mellitus with dyslipidemia.
2. To evaluate the effect of atorvastatin on the lipid profile of patients with type 2 diabetes mellitus with dyslipidemia.

The secondary objective for the study was to compare the safety and efficacy of rosuvastatin with simvastatin and atorvastatin in patients with type 2 diabetes mellitus with dyslipidemia.

## Results

Rosuvastatin, atorvastatin, and simvastatin were very effective in reducing the levels of serum cholesterol, serum triglyceride, LDL, and VLDL after treatment for 12 weeks in patients with type 2 diabetes mellitus with dyslipidemia. The reductions in these lipid parameters were highly significant. All the three statins also increased the levels of HDL significantly (*P* < 0.001) after treatment for 12 weeks [Table 1].

**Table 1:** Comparative effect of rosuvastatin, atorvastatin and simvastatin on lipid profile parameter before and after therapy

Lipid profile parameter (mgs %)	Rosuvastatin Mean ± SD		Atorvastatin Mean ± SD		Simvastatin Mean ± SD	
		
	Before	After	Before	After	Before	After
Serum cholesterol	284.38 ± 50.81	196.71 ± 32.57	270.86 ± 43.32	201.11 ± 33.38	265.19 ± 29.41	217.01 ± 24.06
‘P’ value	< 0.001**	< 0.001**	< 0.001**			
Serum triglyceride	245.46 ± 32.42	196.06 ± 26.94	255.41 ± 45.13	221.84 ± 77.00	228.70 ± 29.37	205.90 ± 27.96
‘P’ value	< 0.001**	< 0.05*	< 0.001**			
HDL	42.06 ± 3.30	49.76 ± 5.04	42.46 ± 7.71	45.48 ± 7.26	39.72 ± 6.87	41.53 ± 7.06
‘P’ value	< 0.001**	< 0.001**	< 0.001**			
LDL	193.23 ± 50.28	107.73 ± 32.87	177.34 ± 46.29	114.27 ± 35.85	179.73 ± 31.21	134.49 ± 26.34
‘P’ value	< 0.001**	< 0.001**	< 0.001**			
VLDL	49.09 ± 6.48	39.21 ± 5.39	51.05 ± 9.03	41.37 ± 8.24	45.74 ± 5.87	40.99 ± 5.71
‘*P*’ value	< 0.001**		< 0.001**		< 0.001**	

HDL: High-density lipoproteins, LDL: Low density lipoproteins, VLDL: Very low density lipoproteins,

** ‘*P*’ <0.001 (Highly statistically significant),

* ‘*P*’< 0.05 (Statistically significant)

There was statistically significant increase in HDL (49.76 ± 5.04 *vs*. 45.48 ± 7.26, *P* < 0.05) levels in rosuvastatin group when compared with atorvastatin after therapy. However, the reductions in serum cholesterol, triglyceride, LDL, and VLDL showed no statistically significant difference in both the groups (*P* > 0.05)** [Table 2].

**Table 2:** Comparative effect of rosuvastatin and atorvastatin on lipid profile parameter after therapy

Lipid profile parameter (mgs %)	After rosuvastatin therapy Mean ± SD	After atorvastatin therapy Mean ± SD	‘*P*’ value
Serum cholesterol	196.71 ± 32.57	201.11 ± 33.38	> 0.05
Serum triglyceride	196.06 ± 26.94	221.84 ± 77.00	> 0.05
HDL	49.76 ± 5.04	45.48 ± 7.26	< 0.05*
LDL	107.73 ± 32.87	114.27 ± 35.85	> 0.05
VLDL	39.21 ± 5.39	41.37 ± 8.24	> 0.05

HDL: High-density lipoproteins,

* ‘*P*’ < 0.05 (Statistically significant), LDL: Low density lipoproteins, VLDL: Very low density lipoproteins.

When compared with simvastatin group, the patients of rosuvastatin group showed statistically significant reduction in serum cholesterol group (196.71 ± 32.57 *vs*. 217.01 ± 24.06, *P* < 0.05) and LDL levels (107.73 ± 32.87 *vs.* 134.49 ± 26.34, *P* < 0.05). The increase in HDL levels in rosuvastatin group was highly significant (49.76 ± 5.04 *vs.* 41.53 ± 7.06, p < 0.001) when compared with simvastatin group after treatment for 12 weeks. Serum triglycerides and VLDL showed no significant difference in both the groups (*P* > 0.05) [Table 3].

**Table 3:** Comparative effect of rosuvastatin and simvastatin on lipid profile parameter after therapy

Lipid profile parameter (mgs %)	After rosuvastatin therapy Mean ± SD	After simvastatin therapy Mean ± SD	‘*P*’ value
Serum cholesterol	196.71 ± 32.57	217.01 ± 24.06	< 0.05*
Serum triglyceride	196.06 ± 26.94	205.90 ± 27.96	> 0.05
HDL	49.76 ± 5.04	41.53 ± 7.06	< 0.001**
LDL	107.73 ± 32.87	134.49 ± 26.34	< 0.05*
VLDL	39.21 ± 5.39	40.99 ± 5.71	> 0.05

Atorvastatin significantly reduced LDL levels (114.27 ± 35.85 vs. 134.49 ± 26.34, P<0.05) as compared to simvastatin but showed no statistically significant difference (P > 0.05) in other studied lipid parameters of type 2 diabetics after treatment [Table 4].

**Table 4:** Comparative effect of atorvastatin and simvastatin on lipid profile parameter after therapy

Lipid profile parameter (mgs %)	After atorvastatin therapy Mean ± SD	After simvastatin therapy Mean ± SD	‘*P*’ value
Serum cholesterol	201.11 ± 33.38	217.01 ± 24.06	> 0.05
Serum triglyceride	221.84 ± 77.00	205.90 ± 27.96	> 0.05
HDL	45.48 ± 7.26	41.53 ± 7.06	> 0.05
LDL	114.27 ± 35.85	134.49 ± 26.34	< 0.05*
VLDL	41.37 ± 8.24	40.99 ± 5.71	> 0.05

HDL: High-density lipoproteins, LDL: Low density lipoproteins, VLDL: Very low density lipoproteins, ‘*P*’ <0.001** (Highly statistically significant),

* ‘*P*’ <0.05* (Statistically significant)

Rosuvastatin reduced LDL levels by 44.25%, atorvastatin reduced LDL levels by 35.56%, and simvastatin reduced LDL levels by 25.17%. Rosuvastatin showed 30.83% reduction in cholesterol levels while atorvastatin and simvastatin reduced cholesterol levels by 25.75 and 18.17% respectively. The HDL levels were increased by 18.31, 7.11, and 4.56% in the rosuvastatin, atorvastatin, and simvastatin groups respectively [Table 5].

**Table 5:** Percentage changes on the various parameters of lipid profile after administration of rosuvastatin, atorvastatin, and simvastatin

Lipid profile parameter (mgs %)	Rosuvastatin group (%)	Atorvastatin group (%)	Simvastatin group (%)
Serum cholesterol	↓ 30.83	↓ 25.75	↓ 18.17
Serum triglyceride	↓ 20.13	↓ 13.14	↓ 9.97
HDL	↑ 18.31	↑ 7.11	↑ 4.56
LDL	↓ 44.25	↓ 35.56	↓ 25.17
VLDL	↓ 20.13	↓ 18.96	↓ 10.38

HDL: High-density lipoproteins, LDL: Low density lipoproteins, VLDL: Very low density lipoproteins

No adverse events were observed in any of the study groups. Rosuvastatin, atorvastatin, and simvastatin group did not deviate significantly from their baseline biochemical profile after 12 weeks of therapy.

## Discussion

Type 2 diabetes is emerging as a major public health problem and seems to occur decade earlier in our country compared to the west. Diabetic care Asian-India study found 40% obesity in urban Indian type 2 diabetes mellitus. They also found inadequate glycemic control and late diabetic complications at the mean duration of one year in over 55 percent of patients.[11]

The evidence that lipid lowering drug treatment (especially statins) significantly reduces cardiovascular risk in diabetic and nondiabetic patients is strong and suggests that diabetic patients benefit more in both primary and secondary prevention.[12]

In the present study, the patients studied were type 2 diabetic patients with dyslipidemia, but having good glycemic control with fixed dose combination of tablet glimepiride + metformin. The criteria for evaluation were lipid profile parameters, namely, serum cholesterol, serum triglyceride, LDL, VLDL, and HDL.

Rosuvastatin decreased the levels of serum cholesterol, serum triglyceride, LDL, VLDL and increased the levels of HDL after therapy for 12 weeks. The difference in the studied lipid parameters after therapy was highly statistically significant (*P* < 0.001). These results are in accordance with the pilot study with rosuvastatin conducted by Gleuck *et al*, at The Cholesterol Centre, Jewish Hospital, Cincinnati, USA.[13]

Atorvastatin and simvastatin also decreased the levels of serum cholesterol, serum triglyceride, LDL, VLDL and increased the levels of HDL after therapy for 12 weeks. The difference in the studied lipid parameters after therapy in both the drug groups was highly statistically significant (*P* < 0.001). These results are in accordance with the studies conducted by Goudevenos *et al*[14] and Lewin *et al*,[15] for the efficacy of atorvastatin and simvastatin in dyslipidemia, respectively.

When the LDL level reduction in rosuvastatin group with that of atorvastatin and simvastatin group was compared, it was observed that the reduction in LDL levels in rosuvastatin group were statistically significant when compared with those of simvastatin group, but were statistically nonsignificant when compared with atorvastatin group. These results are in contrast to a study conducted by Bullano *et al*, which concluded that rosuvastatin was more effective than both atorvastatin and simvastatin in reducing LDL levels significantly.[16]

The comparison of reduction in LDL levels between atorvastatin group and simvastatin group were statistically significant. This result is in accordance to a study conducted by Wu *et al*, which showed that patients treated with atorvastatin had a significantly greater reduction in low-density lipoprotein cholesterol as compared to simvastatin.[17]

The rise in HDL levels in rosuvastatin group after therapy was statistically significant when compared with atorvastatin group and was highly statistically significant when compared with simvastatin group. In contrast to this, the use of rosuvastatin *vs* atorvastatin in type 2 diabetes mellitus (URANUS) study group found that both rosuvastatin and atorvastatin increased HDL-C and decreased TG from baseline to 4 weeks, but there were no statistically significant differences between the groups.[18] The COMETS study (a comparative study with rosuvastatin in subjects with metabolic syndrome) concluded that rosuvastatin increased high-density lipoprotein cholesterol significantly more than atorvastatin.[19] However, the comparison of increase in HDL levels between atorvastatin group and simvastatin group were statistically nonsignificant. This result is in contrast to the study conducted by Hunninghake *et al*, which concluded that simvastatin produced larger increases in HDL-C.[20]

The comparison of the serum cholesterol reduction in rosuvastatin group with that of atorvastatin and simvastatin group revealed that the reduction in serum cholesterol levels in rosuvastatin group were statistically significant when compared with those of simvastatin group but were statistically nonsignificant when compared with atorvastatin group. The comparison of reduction in serum cholesterol levels between atorvastatin group and simvastatin group were statistically nonsignificant.

The intergroup comparison of reduction of serum triglycerides and VLDL after therapy among the rosuvastatin, atorvastatin, and simvastatin groups was statistically nonsignificant (*P*>0.05).

Rosuvastatin reduced LDL levels by 44.25%, atorvastatin reduced LDL levels by 35.56%, and simvastatin reduced LDL levels by 25.17%. These results are consistent with the STELLAR trial where rosuvastatin, atorvastatin, and simvastatin reduced LDL levels by 45.8, 36.8, and 28.3%, respectively.[21]

## Conclusion

In summary, 10 mg of rosuvastatin tablet was comparable to 10 mg of atorvastatin tablet and more efficacious than 10 mg tablet simvastatin in reducing LDL levels after 12 weeks of therapy in patients of type 2 diabetes mellitus with dyslipidemia. Also, 10 mg of rosuvastatin was more efficacious than 10 mg of both atorvastatin and simvastatin in increasing HDL levels after 12 weeks of therapy in patients of type 2 diabetes mellitus with dyslipidemia. No adverse events were noted with any of the three statins used. However, further studies are necessary to conclusively prove the efficacy of rosuvastatin over the existing statins.

## References

[1] Krall LP, Beaser RS (1996). Prologue. Joslins Diabetes Manual.

[2] O'Brien RC, Luo M, Balazs N (2000). *In vitro* and *in vivo* antioxidant properties of Gliclazide. J Diabetes Compl.

[3] Taskinen MR (1998). Strategies for the management of diabetic dyslipidemia. Drugs.

[4] Zimmet P, Collier G (1998). Clinical efficacy of metformin against insulin resistance parameters. Drugs.

[5] Moon YK, Kashyap ML (2004). Pharmacologic treatment of type 2 diabetic dyslipidemia. Pharmacotherapy.

[6] Knopp RH, D'Emden M, Smilde JG, Pocock SJ (2006). Efficacy and safety of atorvastatin in the prevention of cardiovascular end points in subjects with type 2 diabetes. Diabetes Care.

[7] The Diabetes Atorvastatin Lipid Intervention (DALI) Study Group (2001). The Effect of Aggressive Versus Standard Lipid Lowering by Atorvastatin on Diabetic Dyslipidemia. Diabetes Care.

[8] Pedersen TR, Faergeman O, Kastelein JP, Olsson AG, Tikkanen MJ, Holme I (2005). High-dose atorvastatin vs. usual-dose simvastatin for secondary prevention after myocardial infarction. JAMA.

[9] Wierzbicki AS, Lumb P, Semra Y, Chik G, Christ ER, Crook MA (1999). Atorovastatin compared with with simvastatin based therapies in the management of severe familial hyperlipidemias. Q J Med.

[10] Ferdinand KC, Clark LT, Watson KE, Neal RC, Brown CD, Kong BW (2006). Comparison of efficacy and safety of rosuvastatin versus atorvastatin in african-american patients in a six-week trial. Am J Cardiol.

[11] Raman PG (2002). Diabetes mellitus in India: 2001. J Gen Med.

[12] Costa J, Borges M, David C, Carneiro AV (2006). Efficacy of lipid lowering drug treatment for diabetic and non-diabetic patients: Meta-analysis of randomized controlled trials. BMJ.

[13] Glueck CJ, Aregawi D, Agloria M, Khalil Q, Winiarska M, Munjal J (2006). Rosuvastatin 5 and 10 mg/d: A pilot study of the effects in hypercholesterolemic adults unable to tolerate other statins and reach LDL cholesterol goals with nonstatin lipid-lowering therapies. Clin Ther.

[14] Goudevenos JA, Bairaktari ET, Chatzidimou KG, Milionis HJ, Mikhailidis DP, Elisaf MS (2001). The Effect of Atorvastatin on Serum Lipids, Lipoprotein (a) and Plasma Fibrinogen Levels in Primary Dyslipidaemia -- A Pilot Study Involving Serial Sampling. Curr Med Res Opin.

[15] Lewin AJ, Kipnes MS, Meneghini LF, Plotkin DJ, Perevozskaya IT, Shah S (2004). Effects of simvastatin on the lipid profile and attainment of low-density lipoprotein cholesterol goals when added to thiazolidinedione therapy in patients with type 2 diabetes mellitus: A multicenter, randomized, double-blind, placebo-controlled trial. Clin Ther.

[16] Bullano MF, Wertz DA, Yang GW, Kamat S, Borok GM, Gandhi S (2006). Effect of rosuvastatin compared with other statins on lipid levels and National Cholesterol Education Program goal attainment for low-density lipoprotein cholesterol in a usual care setting. Pharmacotherapy.

[17] Wu CC, Sy R, Tanphaichitr V, Hin AT, Suyono S, Lee YT (2002). Comparing the efficacy and safety of atorvastatin and simvastatin in Asians with elevated low-density lipoprotein-cholesterol--a multinational, multicenter, double-blind study. J Formos Med Assoc.

[18] Berne C, Siewert-Delle A (2005). the URANUS study investigators. Comparison of rosuvastatin and atorvastatin for lipid lowering in patients with type 2 diabetes mellitus: results from the URANUS study. Cardiovasc Diabetol.

[19] Stalenhoef AF, Christie M, Ballantyne CM, Sarti C, Murin J, Tonstad S (2005). A Comparative study with rosuvastatin in subjects with Metabolic Syndrome: Results of the COMETS study. Eur Heart J.

[20] Hunninghake DB, Ballantyne CM, Maccubbin DL, Shah AK, Gumbiner B, Mitchel YB (2003). Comparative effects of simvastatin and atorvastatin in hypercholesterolemic patients with characteristics of metabolic syndrome. Clin Ther.

[21] Jones PH, Davidson MH, Stein EA, Bays HE, McKenney JM, Miller E (2003). Comparison of safety and efficacy of Rosuvastatin versus Atorvastatin, Simvastatin and Pravastatin across doses (STELLAR trial). Am J Cardiol.

